# An FDA-Validated, Self-Cleaning Liquid Chromatography–Mass Spectrometry System for Determining Small-Molecule Drugs and Metabolites in Organoid/Organ-on-Chip Medium

**DOI:** 10.1021/acs.analchem.4c02246

**Published:** 2024-07-10

**Authors:** Stian Kogler, Gustav Mathingsdal Pedersen, Felipe Martínez-Ramírez, Aleksandra Aizenshtadt, Mathias Busek, Stefan J. K. Krauss, Steven Ray Wilson, Hanne Røberg-Larsen

**Affiliations:** † Hybrid Technology Hub-Centre of Excellence, Institute of Basic Medical Sciences, Faculty of Medicine, University of Oslo, Oslo 0372, Norway; ‡ Section for Chemical Life Sciences, Department of Chemistry, University of Oslo, Oslo NO-0315, Norway; § Department of Analytical Chemistry, Faculty of Science, Charles University, Prague CZ-128 43, Czech Republic

## Abstract

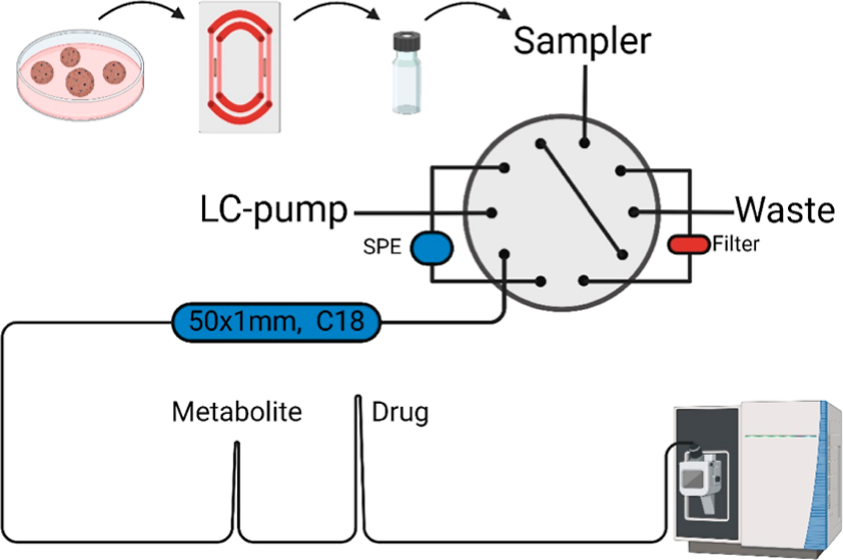

As organoids and organ-on-chip (OoC) systems move toward preclinical and clinical applications, there is an increased need for method validation. Using a liquid chromatography–mass spectrometry (LC–MS)-based approach, we developed a method for measuring small-molecule drugs and metabolites in the cell medium directly sampled from liver organoids/OoC systems. The LC–MS setup was coupled to an automatic filtration and filter flush system with online solid-phase extraction (SPE), allowing for robust and automated sample cleanup/analysis. For the matrix, rich in, e.g., protein, salts, and amino acids, no preinjection sample preparation steps (protein precipitation, SPE, etc.) were necessary. The approach was demonstrated with tolbutamide and its liver metabolite, 4-hydroxytolbutamide (4HT). The method was validated for analysis of cell media of human stem cell-derived liver organoids cultured in static conditions and on a microfluidic platform according to Food and Drug Administration (FDA) guidelines with regards to selectivity, matrix effects, accuracy, precision, etc. The system allows for hundreds of injections without replacing chromatography hardware. In summary, drug/metabolite analysis of organoids/OoCs can be performed robustly with minimal sample preparation.

## Introduction

Organoids can broadly be described as three-dimensional organ models, derived from, e.g., human stem cells.^1^ An organoid may contain several organ-specific cell types and recapitulate corresponding functions. Organ models created in a microfluidic device are called organ-on-chip (OoC).^2^ Organoids and OoC devices are predicted to be key tools in drug testing, disease modeling, and personalized medicine while reducing the use of animal models.^3,4^ The possibilities of these technologies are reflected in legislative changes in the US where drug testing using animals is no longer a mandatory step to receive Food and Drug Administration (FDA) approval of preclinical drug testing.^5^ However, in order to replace animal experimentation in drug development, stringent testing, standardization, and adherence to validation methods and performance criteria will be paramount to strengthen the validity of organoid- and OoC-based models over animal models.^6^

To study drug metabolism in organoids/OoCs, analytical methods for direct measurement of small molecules are required. Mass spectrometry (MS) is a key tool in this context, e.g., MS imaging and liquid chromatography–mass spectrometry (LC–MS).^7^ While the latter is emerging as a powerful tool for mapping the distribution of chemicals within biological tissues, e.g., the organoids themselves, LC–MS is highly suited for the analysis of cell culture medium solutions.

LC–MS can selectively identify and distinguish drugs and structurally similar metabolites, allow for the structural determination of unknown metabolites, and provide precise and accurate quantitative determinations. However, the compelling traits of LC–MS can be obstructed if samples contain interferents, such as highly abundant chemicals that can suppress or enhance the signal of analytes (known as matrix effects). Cell culture medium is a complex matrix containing high amounts of proteins, salts, nutrients, and a plethora of biomolecules and vesicles secreted from the organoids into the medium.^8^ Direct analysis of cell culture medium is possible but can affect column choice and separation performance and increase maintenance, for example, electrospray ionization (ESI) source cleaning.^9^ We therefore see it as paramount to explore sample preparation approaches for LC–MS analysis of cell culture medium samples from organoids/OoCs.

Novel preparation devices for organoid/OoC medium are being investigated, including online electromembrane extraction and preparative gel electrophoresis systems (not yet commercially available).^10,11^ An alternative approach may be to employ online solid-phase extraction (SPE). However, online sample handling is also prone to clogging, so we developed an automatic filtration and filter flush (AFFL) system to prepare biological samples for online SPE–LC–MS analysis.^12^ AFFL is an automated self-cleaning system featuring a microbore filter that is back-flushed postinjection, which allows for online SPE–LC to be performed without pressure buildup. An AFFL setup has recently been used for studying endogenous lipids in organoid samples, following extraction and derivatization steps.^13^

**Figure 1:**
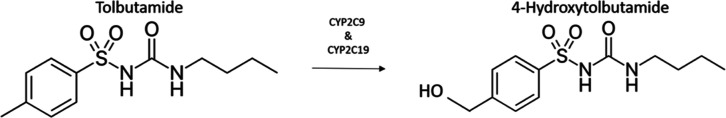
Structure of the antidiabetic drug tolbutamide and its associated metabolite 4-HT which is formed through CYP2C9 and CYP2C19 activity.

Here, we applied AFFL–SPE–LC–MS for detecting small-molecule drugs and drug metabolites in media collected from human stem cell-derived liver organoids^14^ and a liver-on-chip system.^15^ In contrast to the aforementioned lipid methodology,^13^ no preinjection preparation steps for the complex sample matrix are required. Using FDA guidelines,^16^ we demonstrate and validate the applicability of the analytical system through the analysis of the familiar antidiabetic drug tolbutamide and its associated metabolite 4-hydroxytolbutamide (4HT, Figure 1).

## Experimental Section

### Reagents and Solutions

Water (LC–MS grade), methanol (MeOH, LC–MS grade), and acetonitrile (LC–MS grade) were purchased from VWR International (Radnor, PA, USA). Formic acid (FA, ≥98%), tolbutamide (≥98%), and 4HT (≥98%) were purchased from Merck (Darmstadt, Germany). Tolbutamide-*d*_9_ (9-deuterated, ≥99%) was purchased from Cayman Chemicals (Ann Arbor, MI, USA). Stock solutions of the tolbutamide compounds were prepared in acetonitrile (ACN) at 1 mg/mL concentration and stored at −20 °C before use.

Williams’ E medium (Thermo Fischer Scientific) was supplemented with 1% fetal bovine serum (FBS, Thermo Fisher Scientific) or 0.5% bovine serum albumin (BSA, VWR), 1% GlutaMAX (Thermo Fischer Scientific), 0.1% insulin–transferrin–selenium (Thermo Fischer Scientific), 1% minimum essential medium (MEM) non-essential amino acids solution (Thermo Fischer Scientific), and 0.5% penicillin/streptomycin (Thermo Fischer Scientific). For drug exposure experiments with organoids, an FBS-supplemented medium was used (hereafter referred to as the “main medium”).

### Gel Electrophoresis

A sodium dodecyl-sulfate polyacrylamide gel electrophoresis (SDS-PAGE) was performed to show the matrix samples’ complexity. For comparison, six different matrixes were tested: “main medium”, Williams’ E medium + FBS, Dulbecco’s modified Eagle’s medium (DMEM), and DMEM + FBS. Additionally, we included a “main medium” sample from an OoC experiment. A water-based solution of 10 μg/mL BSA was used as a protein identity marker in an additional well. A Page Ruler protein prestained ladder (Invitrogen, Waltham, PA, USA) was used to identify the molecular weight of proteins and to track the progress of the separation. The full SDS-PAGE procedure was performed according to Olsen et al.^8^

### Liver Organoids and Organ-on-Chips

Liver organoids were generated from human-induced pluripotent stem cells (H1 ESC cell line, WiCell, HMGUi001-A, HMGU, and WTC-11, Coriell Institute for Medical Research) as previously described.^14,17^ After the differentiation protocol, liver organoids were cultured in static conditions in 24 well plates and on the pump-less recirculating OoC (rOoC^15^) platform. Organoids, both in static cultivation and an rOoC, were exposed to tolbutamide-supplemented medium (25–40 μM concentrations) for 24 h.

### AFFL System for Organoid and Organ-on-Chip Samples

**Figure 2:**
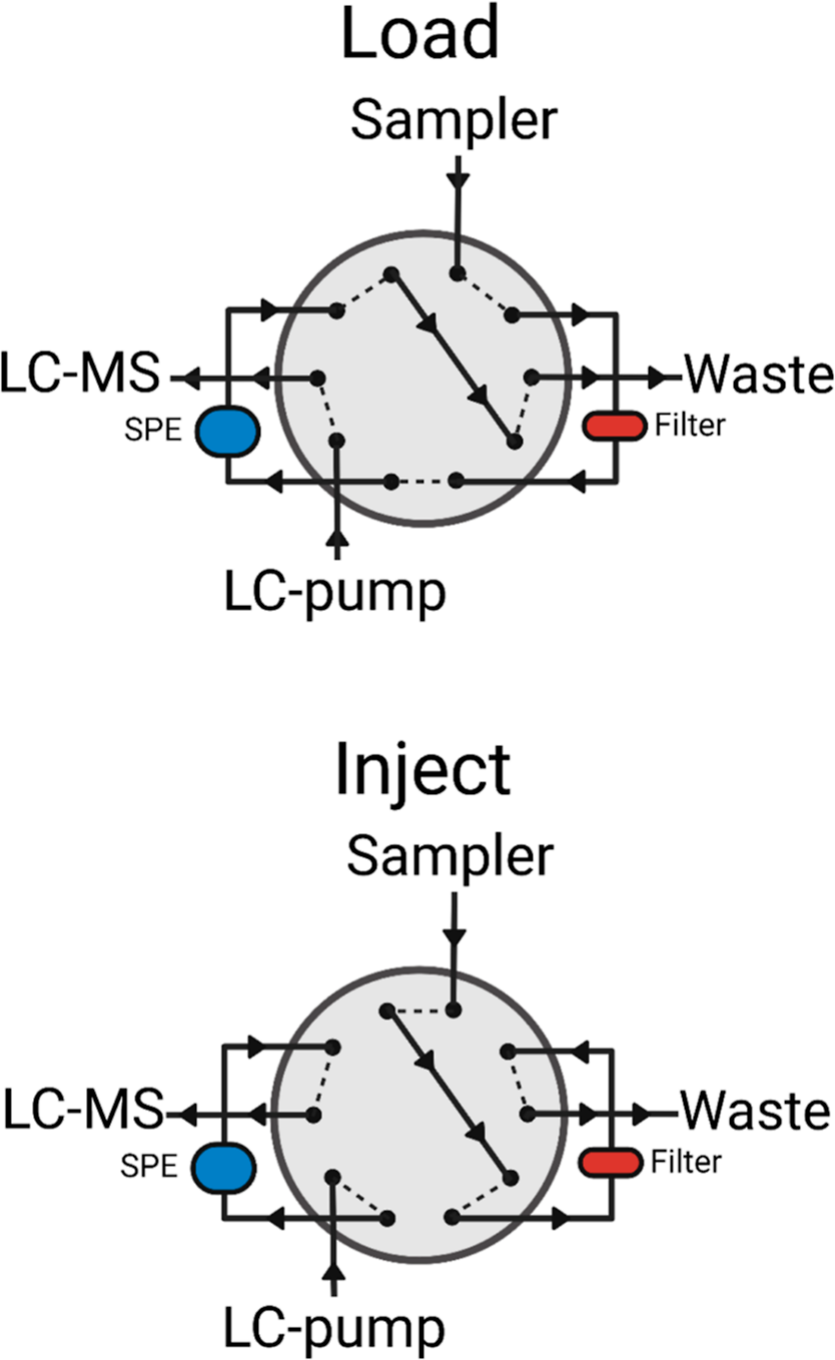
Basic configuration of the AFFL–SPE–LC setup. Cell culture medium is injected directly into the system (after an IS is added). A filter prevents any material that may clog the SPE and the LC column. Following analyte enrichment and removal of polar molecules/salts on the SPE, the analytes are eluted onto the LC column for separation. At the same time, the filter is back-flushed, removing material trapped on the filter.

The AFFL-system plumbing was essentially set up as previously described,^13^ featuring a 1/32″ format switching valve and connections with the LC–MS system, an online reverse-phase SPE column (HotSep Kromasil C18, 1.0 × 5.0 mm, 5 μm, 100 Å), and an online filter unit (VICI, 1 μm pore size). The system was set up in combination with a L-7100 pump (Hitachi High-Technologies, Tokyo, Japan) at a flow rate of 0.100 mL/min used for loading the sample into the AFFL system Figure 2. For SPE loading, the loading pump mobile phase (MP) contained 0.1% FA in a MeOH/water mixture (3%/97%, v/v).

### LC–MS System

The LC–MS system consisted of a Dionex Ultimate 3000 high-performance liquid chromatography apparatus coupled to a TSQ Vantage mass spectrometer with a HESI-II ion source with SST Vipers (130 μm × 650 mm, Thermo Fisher Scientific, Waltham, MA, USA). For separation, a chromatographic column (HotSep Kromasil C18, 1.0 × 50 mm, 3.5 μm, 100 Å, G&T Septech, Ski, Norway) was used.

MP A consisted of 0.1% FA in water (v/v), while MP B consisted of 0.1% FA in MeOH (v/v). A flow rate of 0.100 mL/min was used under gradient conditions (Supporting Information 1). An injection volume of 5 μL was used for all samples, injecting into the AFFL system (AFFL-valve switch at 1.0 min after injection). Subsequently, elution of extracted compounds was performed with the LC pump.

Mass spectrometry detection was carried out after ESI in negative-ion polarity (with polarity switching). All ESI parameters and selected reaction monitoring (SRM) parameters are summarized in Supporting Information 2 and 3.

### Method Validation

The LC–MS method validation was performed following FDA guidelines (M10 Bioanalytical Method Validation and Study Sample Analysis^16^), except for the lower limit of detection (LLOD) and lower limit of quantification (LLOQ), which were calculated using the Eurachem guide.^18^

Tolbutamide and 4HT were used as the main analytes, with tolbutamide-*d*_9_ as an internal standard (IS). The method’s performance was validated for its selectivity, matrix interference, linearity, accuracy, precision, range, LLOD, LLOQ, and carryover for both analytes.

The selectivity was tested by determining the signal interference of six blank replicates of matrix samples without added analytes or IS. The detected signal at the analytes’ retention times (RTs) was compared with the signal from the lowest point of the calibration curve. Matrix effects were evaluated by analyzing the signal from three different media spiked with high (tolbutamide: 30 μM, 4HT: 4000 pM) and low (tolbutamide: 5 μM, 4HT: 300 pM) concentrations of analytes and IS.

The linearity was tested using calibration curves generated with matrix samples at six concentration levels (each level by triplicates, measured three times each). The levels used were 5, 10, 15, 20, 25, and 30 μM for tolbutamide and 300, 500, 800, 1000, 2500, and 4000 pM for 4HT. The “main medium” was used as a diluent in all calibration curves. The linearity of the calibration curves was tested through linear regression describing the concentration–response relationship. The back-calculated concentrations of all calibration standard injections were determined using these curves.

The accuracy and precision of the method were assessed by determining the analytes in QC-samples at low (tolbutamide: 5 μM, 4HT: 300 pM), medium (tolbutamide: 20 μM, 4HT: 1000 pM), and high (tolbutamide: 30 μM, 4HT: 4000 pM) concentration levels with three replicates, injected 6 times each.

The absence of carryover was assessed by analyzing blank matrix samples after the highest concentration level of the calibration curve, injecting three consecutive blanks, and comparing the signals at the analytes’ RTs with the least concentrated calibration standard.

For detailed descriptions of acceptance criteria, sample composition, etc., see Table 1.

**Table 1:** Composition of Various Cell Media^a^

	Williams’ E-W1878	DMEM-D6546	RPMI 1640-R0883	MEM-M0643
components	concentration, g/L			
Inorganic Salts				
calcium chloride	0.2	0.2		0.2
calcium nitrate·4H_2_O			0.1	
cupric sulfate·5H_2_O	0.0000001			
ferric nitrate·9H_2_O	0.0000001	0.0001		
magnesium chloride·4H_2_O	0.0000001		0.04884	
magnesium sulfate (anhydrous)	0.0977	0.09767		0.09767
potassium chloride	0.4	0.4	0.4	0.4
sodium bicarbonate	2.20	3.70	2.00	2.20
sodium chloride	6.80	6.40	6.00	6.80
sodium phosphate monobasic (anhydrous)	0.12	0.109		0.122
sodium phosphate dibasic (anhydrous)			0.8	
zinc sulfate·7H_2_O	0.0000002			
Amino Acids				
l-alanine	0.09			0.0089
l-arginine (free base)	0.05	0.084	0.2	0.126
l-asparagine·H_2_O	0.02		0.05	0.015
l-aspartic acid	0.03		0.02	0.0133
l-cysteine (free acid)	0.04			
l-cystine	0.02	0.0626	0.0652	0.0313
l-glutamic acid	0.0445		0.02	0.0147
l-glutamine	0.292	0.584	0.3	0.292
glycine	0.05	0.03	0.01	0.0075
l-histidine (free base)	0.015	0.042	0.015	0.042
hydroxy-l-proline			0.02	
l-isoleucine	0.05	0.105	0.05	0.052
l-leucine	0.075	0.105	0.05	0.052
l-lysine·HCl	0.08746	0.146	0.04	0.0725
l-methionine	0.015	0.03	0.015	0.015
l-phenylalanine	0.025	0.066	0.015	0.032
l-proline	0.03		0.02	0.0115
l-serine	0.01	0.042	0.03	0.0105
l-threonine	0.04	0.095	0.02	0.048
l-tryptophan	0.01	0.016	0.005	0.01
l-tyrosine·2Na·2H_2_O	0.05045	0.10379	0.02883	0.0519
l-valine	0.05	0.094	0.02	0.046
Vitamins				
ascorbic acid·Na	0.00227			
d-biotin	0.0005		0.0002	
calciferol	0.0001			
choline chloride	0.0015	0.004	0.003	0.001
folic acid	0.001	0.004	0.001	0.001
myo-inositol	0.002	0.0072	0.035	0.002
menadione (sodium bisulfite)	0.00001			
niacinamide	0.001	0.004	0.001	0.001
*p*-aminobenzoic acid			0.001	
d-pantothenic acid (hemicalcium)	0.001	0.004	0.00025	0.001
pyridoxal·HCl	0.001			0.001
pyridoxine·HCl		0.004	0.001	
retinol acetate	0.0001			
riboflavin	0.0001	0.0004	0.0002	0.0001
thiamine·HCl	0.001	0.004	0.001	0.001
dl-α-tocopherol phosphate·Na	0.00001			
vitamin B12	0.002		0.000005	
Other				
d-glucose	2.00	4.51	2.00	1.00
glutathione (reduced)	0.00005		0.001	
methyl linoleate	0.00003			
phenol red·Na		0.0159	0.0053	0.011
pyruvic acid·Na	0.025	0.11		

a Concentrations are shown in g/L. In this study, Williams’ E medium with additives is used. Medium formulations are compiled from the webpages of the producer.

## Results and Discussion

### Description and Visualization of Cell Culture Medium

**Figure 3:**
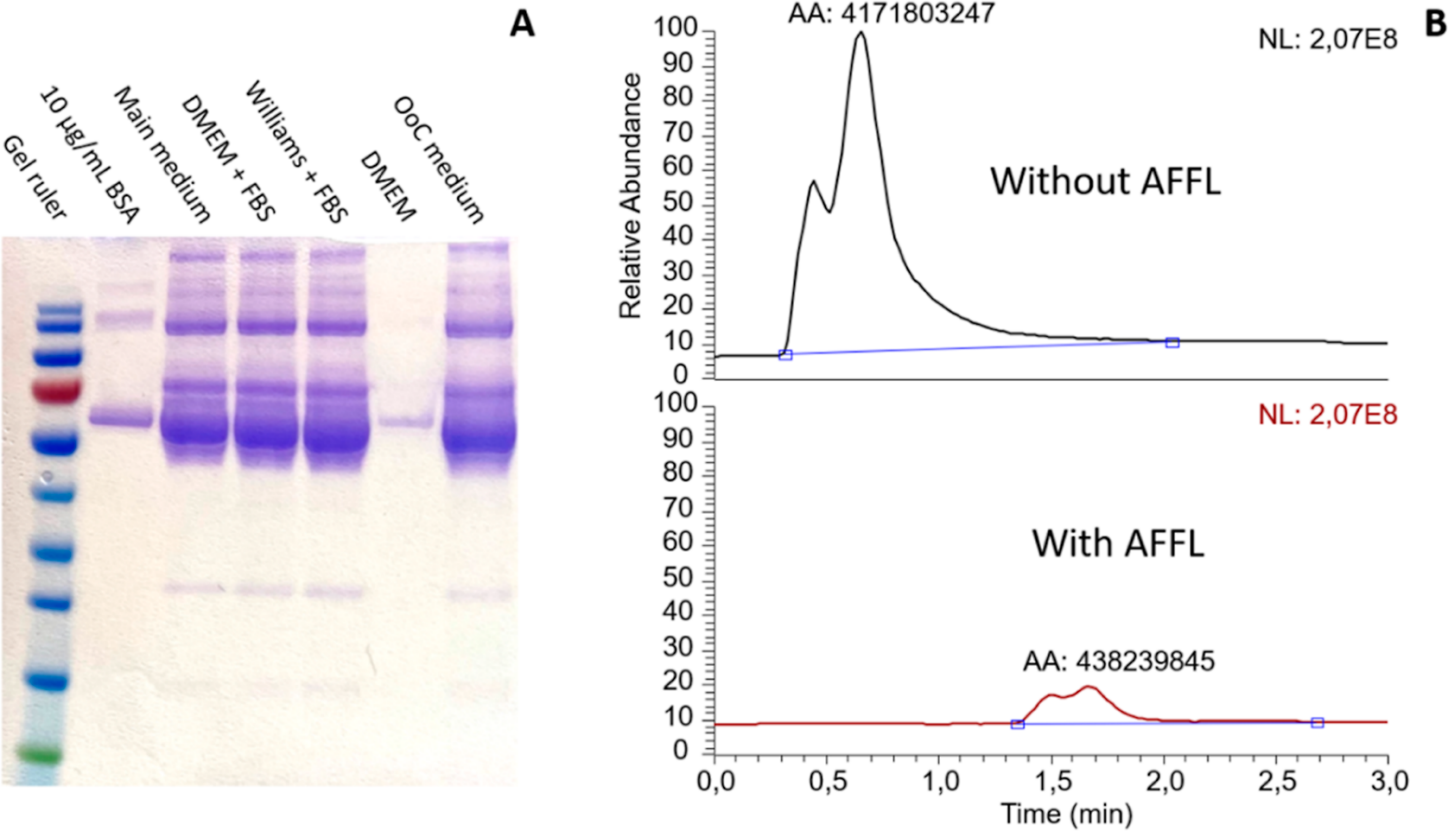
(A) SDS-PAGE of typical mediums, BSA, and an OoC sample. (B) Direct injection of the “main medium” with and without AFFL on the mass spectrometer. The AFFL system reduces the signal of unspecified analytes by ≈90%. The shift in RT is due to 1 min loading/washing time on the AFFL system.

The SDS-PAGE visualizes the complex protein mixture of medium samples (see Figure 3A). The protein found at ≈66 kDa corresponds to albumin, the most abundant protein across the samples. In addition, the cell mediums contain a range of amino acids, salts, and metabolites (not visible in the SDS-PAGE) that further complexify the cell medium as an analytical matrix (see Table 1).

### Method Development

#### AFFL–SPE–LC Details and Considerations

To address the possible interference caused by the complex media for drug/metabolite analysis, we utilized the AFFL–SPE system. In a previous study, the cell culture medium from liver organoid was mixed with ethanol for the preparation of hydrophobic sterols,^13^ which also provided a sample cleanup prior to injection (protein removal after precipitation due to ethanol). Here, we explore an AFFL-based analysis of completely untreated medium samples.

During the initial SPE method development, we utilized a loading MP with 0% organic modifier, as suggested as possible by the SPE manufacturer. However, this was associated with low signal strength and poor repeatability of drug/metabolite detection. Turning to a loading MP of 3% MeOH in water (v/v), we could achieve an ample and reproducible signal of the injected analytes after elution to the separation column with the LC mobile phase. Under these conditions, AFFL–SPE facilitated sample cleanup, resulting in an overall reduction of approximately 90% in the integrated total ion chromatogram area of untargeted analytes (*n* = 3, Figure 3B).

The MS triple quadrupole instrument used in this study has limitations in resolution and sensitivity for detecting “unwanted” macromolecules like albumin. However, there is a substantially reduced need for washing the MS-inlet of biochemical matrix compared to our previous method,^9^ implying that samples cleaned with the AFFL system were of much higher purity when entering the LC–MS system. In addition, when coupled to LC–UV (280 nm), no obvious traces of protein absorbance were recorded. With the conditions used here, pressure buildup on the SPE was not observed. Factors may include the narrow pore size of the SPE material and MP composition, which is somewhat stronger than most of our previous loading solvents. In summary, the AFFL–SPE system allowed for simple extraction of analytes from medium with little maintenance.

#### LC–MS Analysis

Separation of tolbutamide and 4HT was achieved using isocratic elution. With 50% of MP B, symmetric peaks were resolved with RTs of 2.0 for 4HT and 3.7 for tolbutamide (see Supporting Information 4). To further ensure robustness and repeatability of the method, a 2 min ramp was added (to 80% MP B) and a 4 min isocratic wash at 80% MP B with a subsequent re-equilibration of 5 min. The complete method lasted 15 min.

MS conditions were optimized via direct injection of tolbutamide and 4HT in 50% MeOH in water with the MS-control software in negative mode. The proposed spray voltage of 3000 V led to a corona discharge in the ESI source, resulting in falsely high signal intensities and oxidation of the drug. Therefore, the spray voltage was reduced to 2300 V, which gave the highest signal intensities without discharge and oxidation.

The IS was added to all samples at a concentration of 10 μM to correct for possible matrix differences due to biological variations in organoids, possible extraction variations, ion suppression/enhancement in the ESI, etc.

#### Method Validation

Sample description, validation criteria, and obtained validation data are summarized in Table 2.

**Table 2:** Validation Criteria, Sample Composition, and Calculated Values^a^

characteristic	sample description	*n*	acceptance criteria	calculated		
selectivity	blank matrix samples without added analytes or IS	3 days, 6 replicates	response from interfering compounds <20% of the lowest calibration standard for analytes and <5% of the IS response at a given RT	tolbutamide: RT_3.7 min_: 0.007%		
				4-hydroxy tolbutamide: RT_2.0 min_: 4%		
				tolbutamide-*d*_9_: RT_3.6 min_: 0.0005%		
matrix effect	low and high levels of analytes in three different matrixes	3 days, 3 different matrixes at high and low concentrations	for each matrix and level	tolbutamide		
	M1: Williams’ E + 1% FBS, 1% GlutaMAX, 1% NEAA, 0.1% ITS, 0.1% P/S		relative error ±15% of nominal value		rel. err	RSD
	M2: Williams’ E + 1% BSA, 1% Glutamax, 1% NEAA, 0.1% ITS, 0.1% P/S		RSD < 15%	M1H	0.4%	0.6%
	M3: Williams’ E + 1% Glutamax			M1L	0.3%	2%
				M2H	–6%	1%
				M2L	–5%	2%
				M3H	–2%	0.6%
				N3L	–3%	0.8%
				4-hydroxy tolbutamide		
					rel. err	RSD
				M1H	2%	7%
				M1L	1%	5%
				M2H	–5%	6%
				M2L	–6%	10%
				M3H	8%	12%
				M3L	0.8%	5%
calibration curve linearity	spiked medium samples	3 days, 6 levels, each measured 3 times	linearity calculated by the correlation coefficient from the calibration curve	tolbutamide: *R*^2^: 0.9994		
				4-hydroxy tolbutamide: *R*^2^: 0.997		
calibration curve and range	spiked medium samples	3 days, 6 levels, each measured 3 times	back-calculated concentration within ±15% of the nominal value, calibration curve parameters reported, range reported	all back-calculated concentrations of calibration standards were within ±15% of the nominal concentration		
				*X* = concentration (in μmol/L for tolbutamide, in pmol/L for 4HT)		
				*Y* = area_Analyte_/area_IS_		
				*T*: *y* = 0.0694951211*x* – 0.0010398358		
				4HT: *y* = 0.0000002067*x* – 0.0000199579		
				range		
				*T*: 5.0–30.0 μmol/L		
				4HT: 300–4000 pmol/L		
accuracy	QC samples in the sample matrix at low, medium, and high concentration levels	3 days, 5 replicates for low, medium, and high levels	<15% relative error at each level	tolbutamide		
				low: 0.8%		
				medium: –0.4%		
				high: 0.5%		
				4HT		
				low: –2%		
				medium: –1%		
				high: 0.4%		
precision	QC samples in the sample matrix at low, medium, and high concentration levels	3 days, 5 replicates for low, medium, and high levels	<15% RSD within and between day variation	tolbutamide		
				low: 2%		
				medium: 1%		
				high: 0.8%		
				4HT		
				low: 12%		
				medium: 4%		
				high: 5%		
lower limit of detection and quantification	calculated from the lowest calibration solutions			tolbutamide		
				LLOD: 0.06 μM		
				LLOQ: 0.20 μM		
				4HT		
				LLOD: 17 pM		
				LLOQ: 58 pM		
				in practice, the LLOQ is set to the concentration of the lowest calibration standard		
carryover	blank matrix samples (*n* = 3) injected after the highest calibration standard	3 days, 3 replicates	<20% of the analyte response in C1 and <5% of the IS response	tolbutamide: 0.04%		
				4HT: 3%		
				tolbutamide-*d*_9_: 0.01%		

a All matrices are represented by the “main medium” (M1 under matrix effect) unless otherwise stated.

The selectivity test (i.e., background signal at a given RT in a blank sample) showed an interference of 0.007% (tolbutamide) and 4.4% (4HT) when compared to the signal obtained from the lowest calibration solution, which is well below the acceptance criteria (20% interference). Interference at the RT of the IS was measured at 0.0005% and reflects the selectivity of the LC–MS/MS method (see chromatograms in Supporting Information 5).

The matrix effect was evaluated by comparing Williams’ E medium, Williams’ E medium with BSA and other supplements, and Williams’ E medium with FBS and other supplements (“main medium”) spiked with the analytes as described in the Experimental Section and quantified with a calibration curve using the “main medium” as a diluent. None of the three analyzed matrices were found to surpass the acceptance criteria (<15% relative error and relative standard deviation); see chromatograms in Supporting Information 6.

Regarding the linearity of the method, a linear regression was used on all calibration solution runs (*n* = 3 for 6 calibration levels, “main medium” as a matrix). The resulting calibration curves used for determining the analytes were linear (*R*^2^ = 0.99) for both tolbutamide (5–30 μM) and 4HT (0.3–10 nM, see curves in Supporting Information 7). All injections of calibration solutions’ concentrations of analytes were back-calculated and quantified within 15% of the nominal concentration.

The accuracy and precision of the method were examined using spiked samples (tolbutamide: 5, 20, and 30 μM; 4HT: 0.3, 1.0, and 4.0 nM) of the “main medium”. The validation results for both analytes were within the acceptance criteria (<15% relative error and <15% standard deviation), indicating a reproducible and selective determination of tolbutamide and 4HT.

The calculated LLOD was 60 nM for tolbutamide and 17 pM for 4HT. The LLOQ was calculated to be 0.200 nM for tolbutamide and 58 pM for 4HT, which confirmed that the analytes in all samples were accurately quantified. See the Application section below for examples of chromatograms from organoid and OoC samples.

A carryover for both analytes and the IS of ≤3% was measured following injection of the highest calibration solution (well below the acceptance criteria of 20% for analytes and 5% for the IS, see chromatogram in Supporting Information 8).

Our method passed all criteria set for the validation tests, resulting in an accurate, sensitive, and precise LC–MS/MS method that can be used to determine tolbutamide and its metabolite 4HT in organoid medium without any manual preinjection sample preparation using the AFFL system.

#### Application: Tolbutamide Metabolism by Liver Organoids

**Figure 4:**
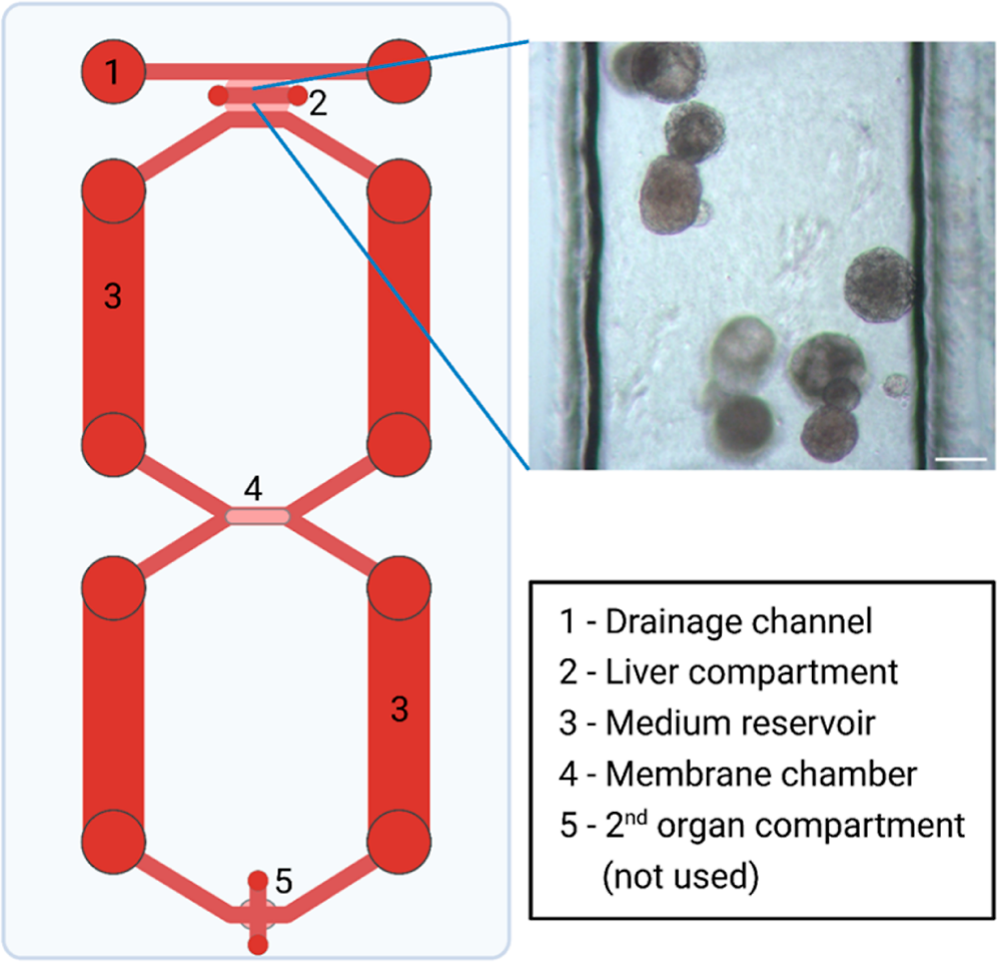
Schematic representation of the rOoC platform used for drug exposure experiments. In the brightfield image, a representative view of liver organoids is shown “on-chip” in the liver compartment of the chip.

**Figure 5:**
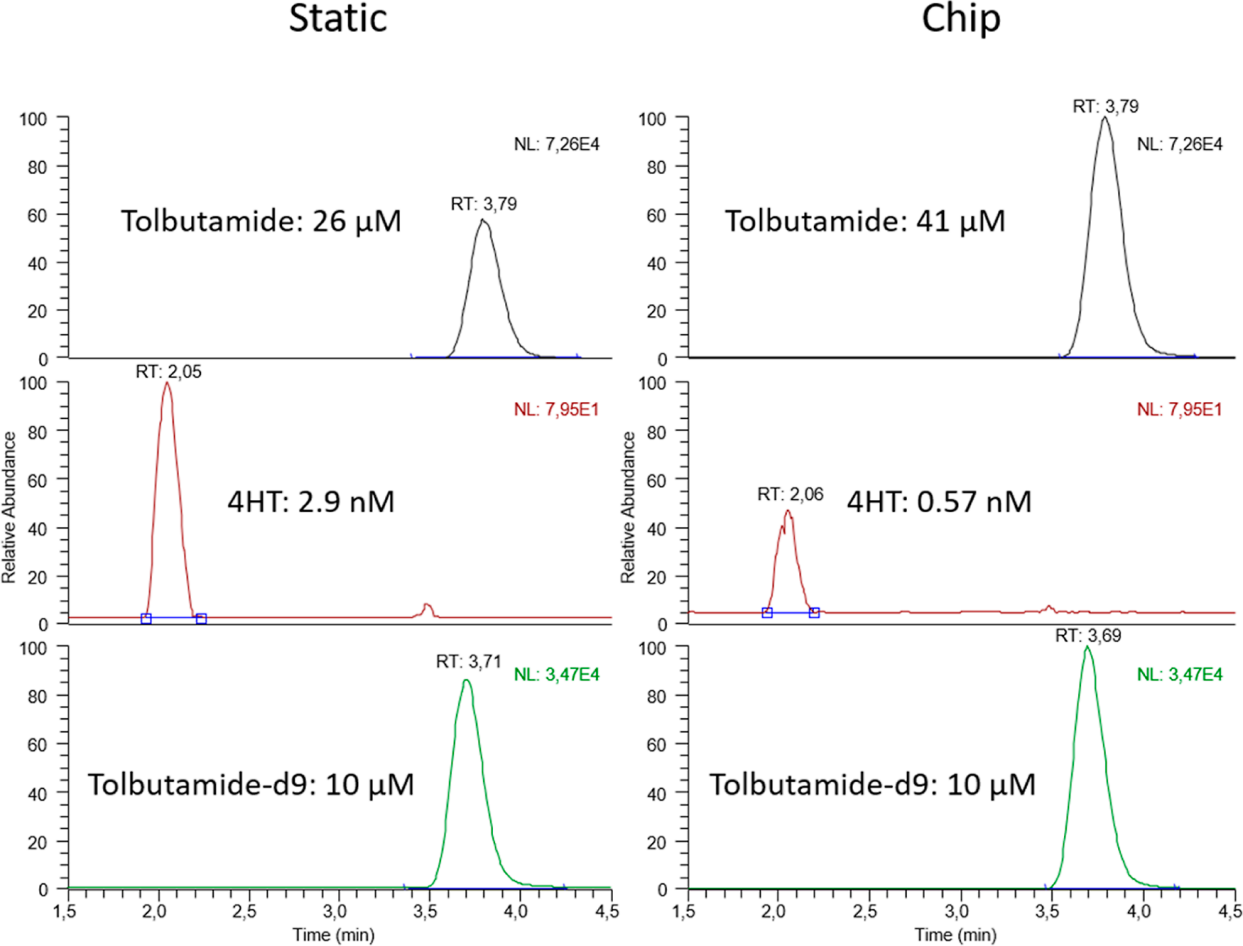
Representative chromatograms of each analyte in medium samples from static and OoC exposure experiments normalized to the highest peak of each analyte (*t* = 24 h).

**Figure 6:**
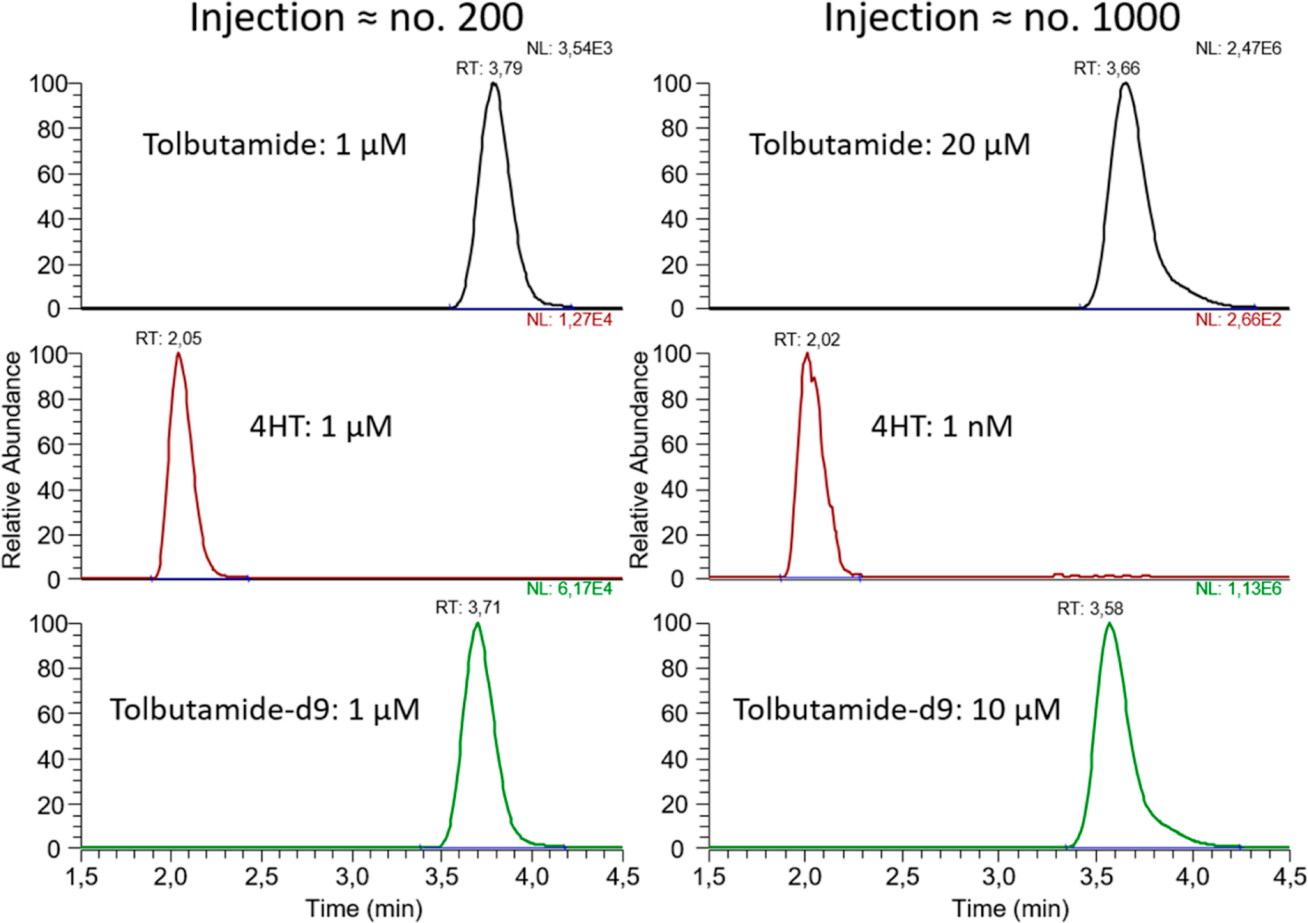
Comparison of SRM chromatograms from an early injection (≈200th injection) and one of the last injections (≈1000th injection) showing no large shift in RTs or loss of peak shape.

We applied our validated method to quantify the amount of 4HT in liver organoid medium after 24 h of incubation of liver organoids (made from stem cell–cell lines H1, HMGUi001-A, and WTC-11) with tolbutamide. Using this method, we were able to analyze samples from liver organoids, both under static conditions (24-well plates) and within a recently developed OoC platform (dual-rOoC, Figure 4). Representative examples of chromatograms for both static and chip exposure experiments are shown in Figure 5. The chromatograms show detection of pM/low nM levels (0.5–3.5 nM) of the metabolite generated by the organoids, which are within the defined quantification range for the validated method (see Supporting Information 9). Any matrix contributions from the organoids (e.g., additional secreted proteins) did not affect the performance of the system, e.g., pressure buildup or shift in RTs. As of today, we have injected over 1000 samples into the system without replacing the AFFL filter, SPE column, and LC column. See Figure 6 for a comparison of injections ≈ nos. 200 and 1000.

## Conclusions

Following regulatory guidelines, we have demonstrated the development and validation of a method for studying drugs and drug metabolism in media from liver organoids and a liver-on-chip platform using a self-cleaning LC–MS platform. The method passed acceptance criteria from FDA guidelines for the validation of bioanalytical methods. The self-cleaning AFFL–SPE feature made preinjection preparation of the complex samples seemingly unnecessary as ca. 1000 samples have been injected without column replacement, showing stable performance. The system was evaluated with a single pair of molecules (tolbutamide and 4HT), but we expect that a significant number of other drugs/metabolites well-matched with RPLC will also be compatible with our general setup. On the same note, we also expect the system to be compatible with alternative RPLC solvents and additives. We are currently establishing a complementary methodology for more polar molecules in cell culture medium using hydrophilic interaction LC (HILIC). We will be investigating downscaling/miniaturization of the system to reduce the environmental impact and increase the sensitivity of minute samples.

## Supporting Information Available

LC, ESI, and MS/MS parameters; validation data; and concentrations of tolbutamide and 4HT in static and OoC conditions (PDF)
