# Supplementary material for: An FDA-Validated, Self-Cleaning Liquid Chromatography–Mass Spectrometry System for Determining Small-Molecule Drugs and Metabolites in Organoid/Organ-on-Chip Medium
Source: Anal Chem. 2024 Jul 10;96(29):12129–38. doi: 10.1021/acs.analchem.4c02246 (PMC11270525; doi:10.1021/acs.analchem.4c02246)
Supplement: Supplementary file 1 — ac4c02246_si_001.pdf [file ac4c02246_si_001.pdf]

Supporting information for:

An FDA validated, self-cleaning liquid chromatography-mass spectrometry system for determining small molecule drugs and metabolites in organoid/organ-on-chip medium

Stian Kogler[1,2], Gustav Mathingsdal Pedersen[2], Felipe Martínez-Ramírez[3], Aleksandra Aizenshtadt[1], Mathias Busek[1], Stefan J. K. Krauss[1], Steven Ray Wilson[1,2]*, Hanne Røberg-Larsen[1,2]

[1] Hybrid Technology Hub - Centre of Excellence, Institute of Basic Medical Sciences, Faculty of Medicine, University of Oslo, Oslo, 0372 Norway

[2] Section for Chemical Life Sciences, Department of Chemistry, University of Oslo, NO-0315 Oslo, Norway

[3] Department of Analytical Chemistry, Faculty of Science, Charles University, CZ-128 43 Prague, Czech Republic

*Corresponding author: Steven Ray Wilson, stevenw@kjemi.uio.no, +47 97010953. Full address: Department of Chemistry, University of Oslo, Post Box 1033, Blindern, NO-0315 Oslo, Norway. https://orcid.org/0000-0002-9755-1188

Table of Content:

Parameters……………………..S2

Validation data……………...S3

Measurements………………S7

Parameters

Supporting Information 1. Liquid chromatography gradient parameters and valve switching times.

Time	%B	Purpose
0-1 min	-	Load sample onto AFFL
1 min	-	AFFL-valve switch to inject position
1-4 min	50	Separation
4-6 min	50-80	Wash
6-10 min	80	
10-12 min	50	Re-equilibration
12 min	-	AFFL-valve switch to load position
12-15 min	50	Re-equilibration

Supporting Information 1. Electrospray ionization parameters.

Parameter	Value
Capillary temperature, °C	380
Vaporizer temperature, °C	350
Sheath gas pressure, arb	45
Aux gas pressure, arb	15
Ion sweep gas pressure, arb	1
Positive spray voltage	2300
Negative spray voltage	2300

Supporting Information 3. MS/MS parameters.

Compound	Parent m/z	Product m/z	Collision Energy, eV	S-Lens	Polarity
Tolbutamide	269.035	105.810	36	80	Neg
		169.705	19	80	Neg
4HT	285.090	103.826	32	66	Neg
		185.759	20	66	Neg
Tolbutamide-d9	278.086	105.810	33	82	Neg
		169.705	21	82	Neg

Validation data

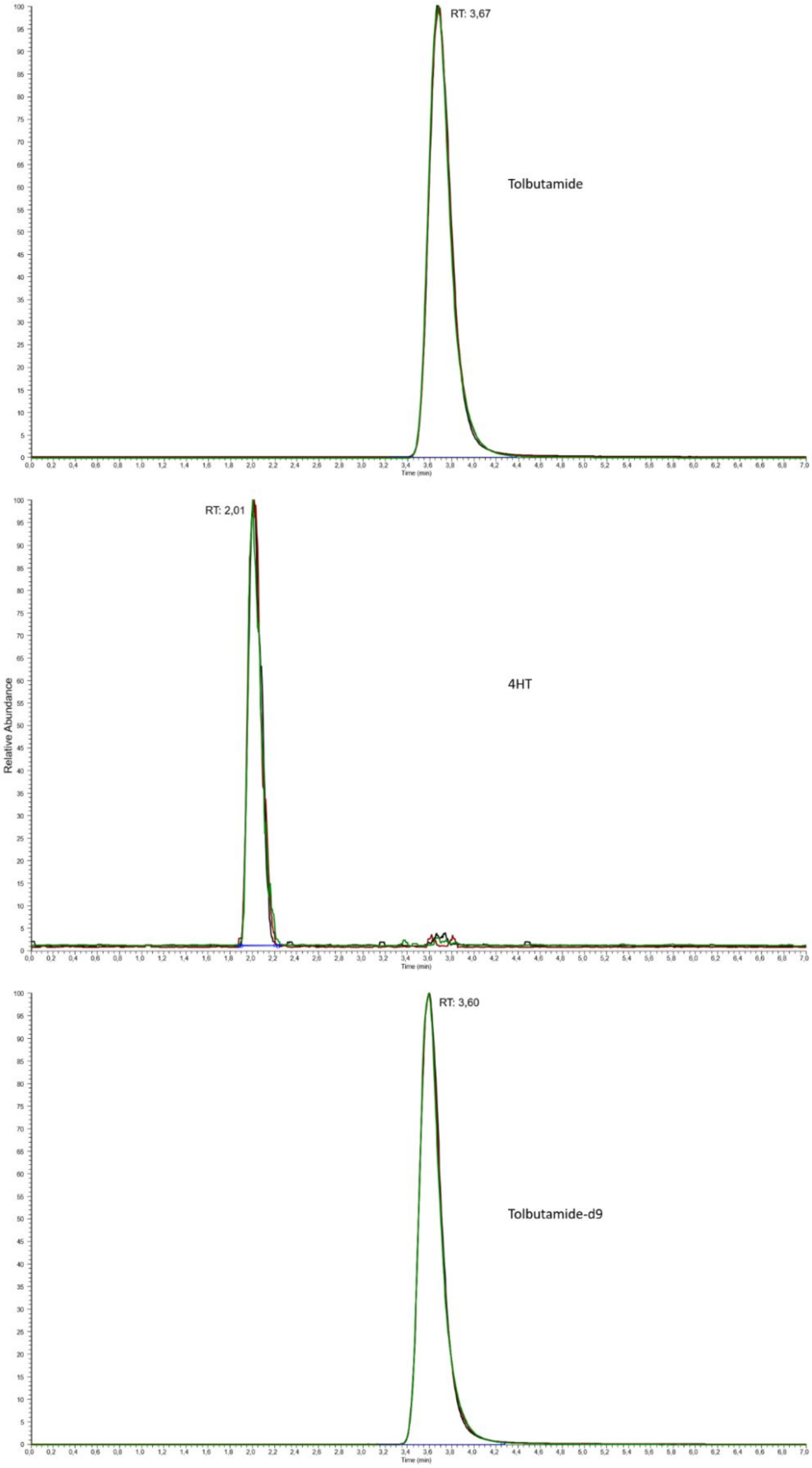

Supporting Information 4. Overlay of three representative chromatograms, each from a different day and sample, showing stable retention times.

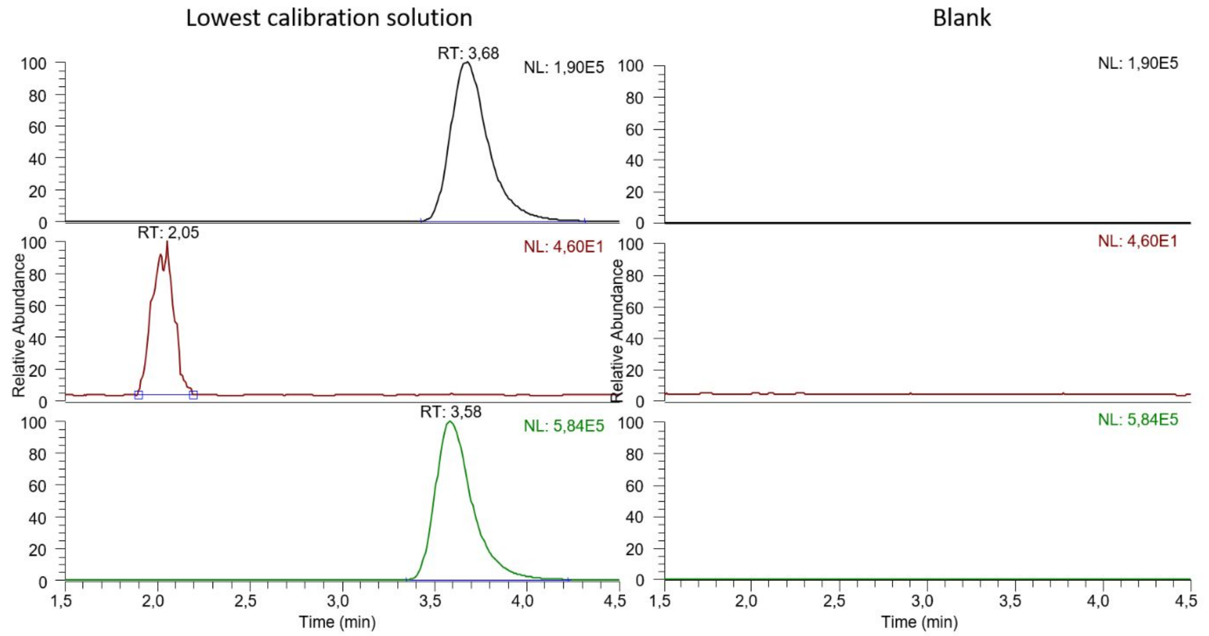

Supporting Information 5. Representative chromatograms of the lowest calibration solution signal shown against the signal from the blanks.

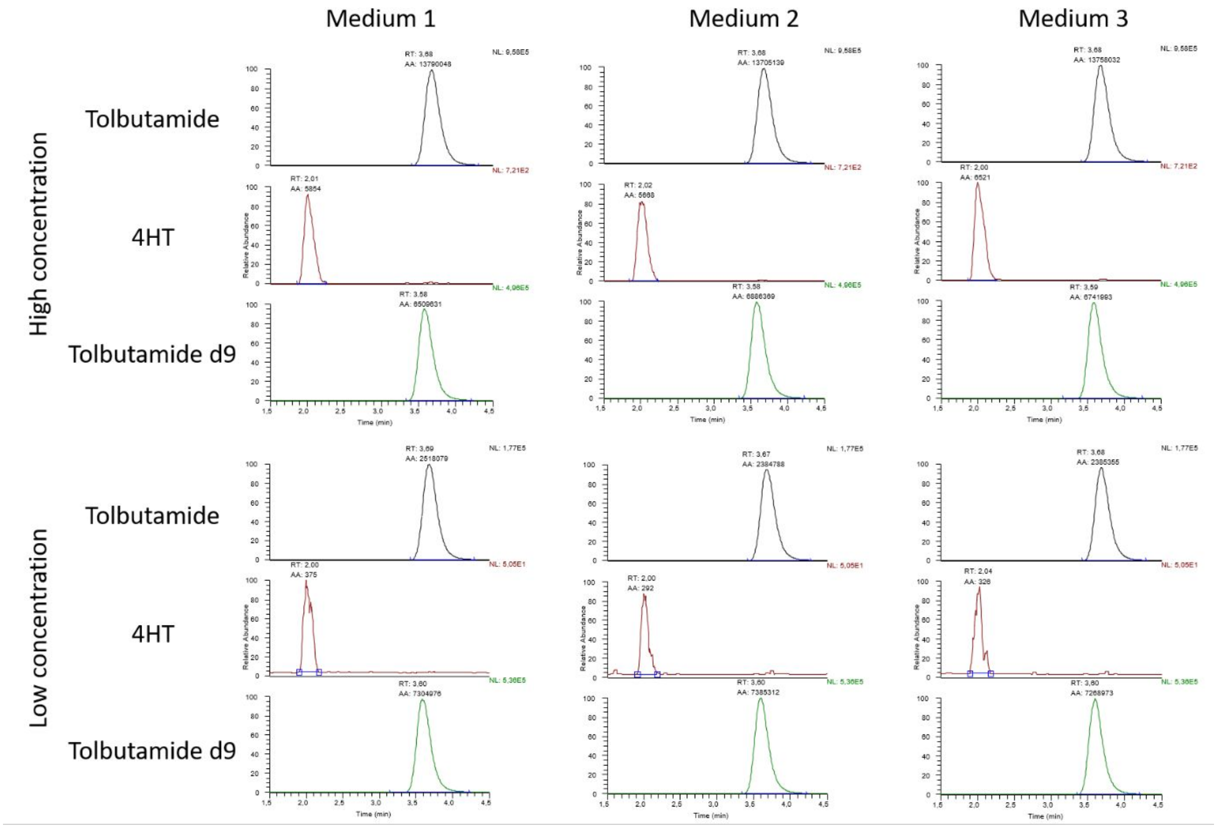

Supporting Information 6. Representative chromatograms of low and high concentrations of tolbutamide and 4HT contained in three different matrices.

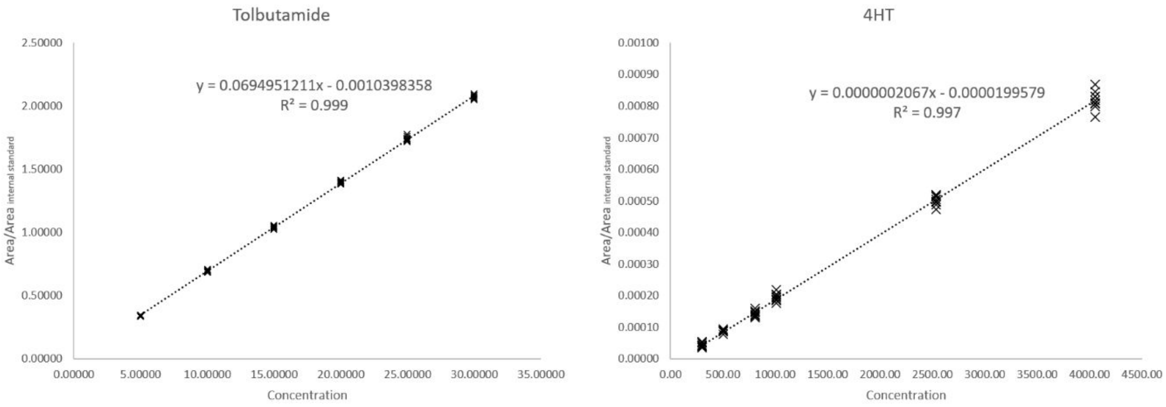

Supporting Information 7. Calibration curves showing the linear trend for each of the analytes of interest.

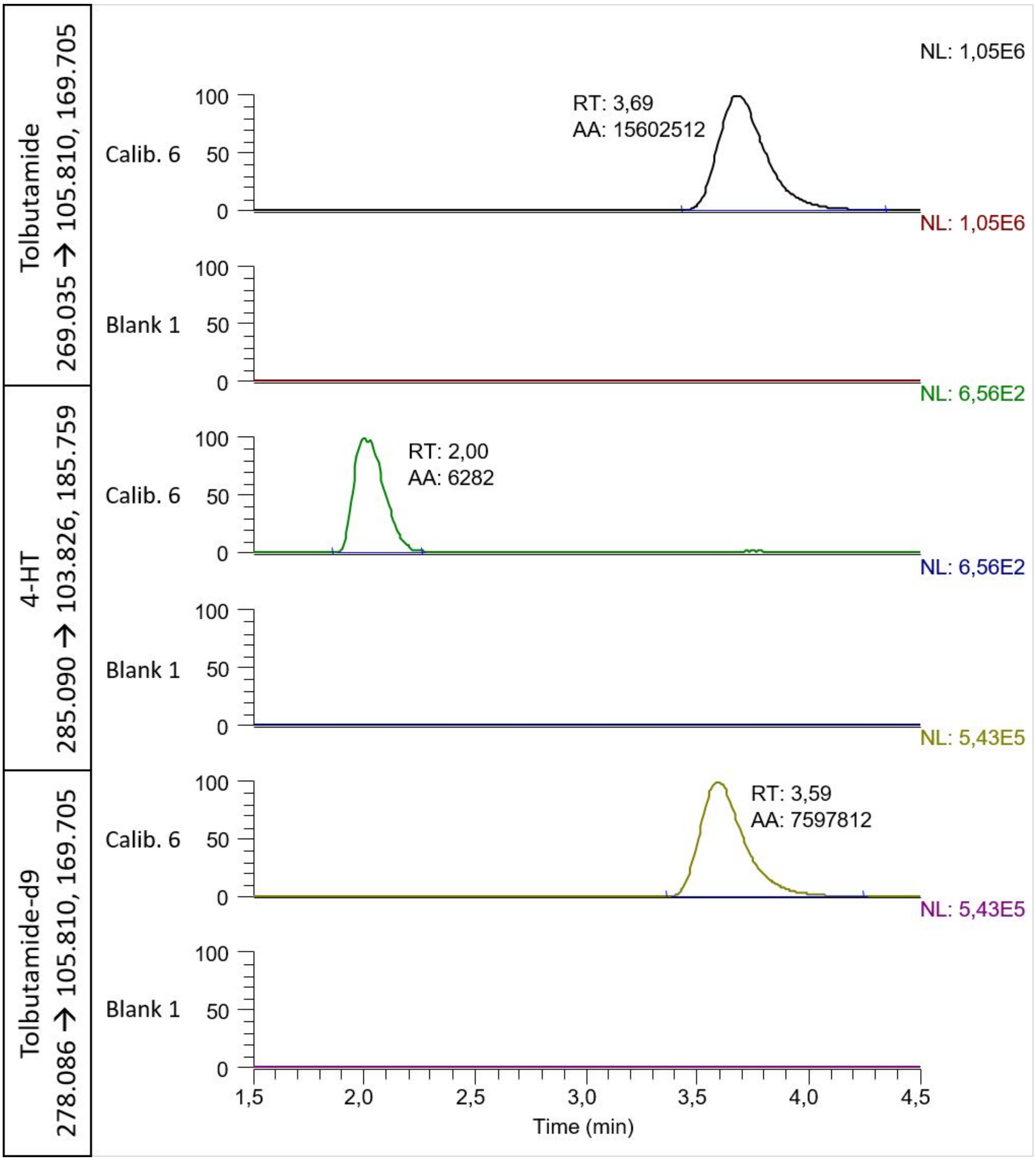

Supporting Information 8. Chromatograms showing a representative injection of the highest calibration solution (up) and the subsequent injection of a blank matrix sample (down) per each analyte.

Measurements

Supporting Information 9. Measured concentrations of tolbutamide and 4HT in static and OoC conditions, represented by different cell origin.

Type	Origin	Replicate	Conc. of Tolbut., µM	AVG, µM	SD, µM	RSD, %	Conc. of 4HT, nM	AVG, nM	SD, nM	RSD, %
Static	H1	1	26.72	26.8	0.3	1.2	1.61	1.7	0.1	7.4
		2	27.16				1.70			
		3	26.51				1.87			
	XH001	1	24.018	23.98	0.03	0.1	1.25	1.1	0.1	10.8
		2	23.951				1.01			
		3	23.979				1.20			
	WTC	1	25.95	25.8	0.03	1.2	3.46	3.1	0.35	11.5
		2	25.93				2.88			
		3	25.39				2.83			
Chip	H1	1	37.389	37.36	0.09	0.2	0.519	0.50	0.01	2.6
		2	37.262				0.493			
		3	37.429				0.500			
	XH001	1	40.99	41.5	0.7	1.7	0.532	0.55	0.02	3.9
		2	41.32				0.534			
		3	42.32				0.570			
	WTC	1	40.89	40.8	0.3	0.7	0.437	0.49	0.07	14.6
		2	41.10				0.569			
		3	40.54				0.457			
